# Methotrexate in chronic-recurrent calcium pyrophosphate deposition disease: no significant effect in a randomized crossover trial

**DOI:** 10.1186/s13075-014-0458-4

**Published:** 2014-10-15

**Authors:** Axel Finckh, Geraldine M Mc Carthy, Anne Madigan, Daniel Van Linthoudt, Marcel Weber, David Neto, Georges Rappoport, Sandra Blumhardt, Diego Kyburz, Pierre-Andre Guerne

**Affiliations:** Division of Rheumatology, University Hospital of Geneva, 26, Ave Beau-Sejour, CH 1211 Geneva, Switzerland; Mater Misericordiae University Hospital, Eccles Street, Dublin 7, Ireland; La Chaux-de-Fonds Hospital, Rue de Chasseral 20, 2300 La Chaux-de-Fonds, Switzerland; Triemli Hospital, Birmensdorferstrasse 497, 8063 Zurich, Switzerland; Yverdon Hospital, Entremonts 11, 1400 Yverdon-les-Bains, Switzerland; Basel University Hospital, Petersplatz 1, 4003 Basel, Switzerland

## Abstract

**Introduction:**

Calcium pyrophosphate deposition (CPPD) may cause severe arthropathy, major joint destruction and treatment options are limited. The aim of this study was to test the therapeutic efficacy of methotrexate (MTX) in chronic or recurrent CPPD arthropathy.

**Methods:**

Patients with CPPD arthropathy were randomized to receive either weekly subcutaneous injections of 15 mg/week of MTX or placebo (PBO) for three months, in a double-blind, crossover randomized controlled trial. Inclusion criteria comprised definite CPPD disease, recurrent arthritis or persistent polyarthritis, and an insufficient response to NSAIDs, glucocorticoids or colchicine. The primary outcome was an improvement in the disease activity scores based on 44 joints (DAS44). The analysis was performed on an intent-to-treat basis.

**Results:**

We randomized 26 patients, and compared 25 treatment periods on MTX with 21 treatment periods on PBO. Baseline characteristics were balanced between the groups. The evolution of the DAS44 was not statistically significantly different between groups (median DAS44 decreased by −0.08 on MTX versus −0.13 on PBO, after three months, *P* = 0.44). Furthermore, pain levels remained stable in both groups (median change in VAS Pain −1 unit on MTX and 0 on PBO, *P* = 0.43), and none of the secondary outcomes was significantly different between the two groups. Minor adverse events (AE) did not differ in frequency between the groups, but the only serious AE occurred on MTX (bicytopenia).

**Conclusions:**

The results of this trial with MTX in this older population with chronic or recurrent CPPD arthropathy suggest no strong effect of MTX on disease activity.

**Trial registration:**

EudraCT No: 2007-003479-37. Registered 26 April 2008

## Introduction

Calcium pyrophosphate deposition (CPPD) disease is a very common and occasionally severe arthropathy associated with chondrocalcinosis. Chondrocalcinosis is itself a metabolic condition due to CPPD, particularly frequent in the second half of life. It can be asymptomatic, at least at the beginning, but various forms of arthropathies develop in affected individuals. Symptomatic presentations include acute CPP arthritis, sometimes called pseudogout, and a chronic arthropathy, generally recurrent, associating osteoarthritis (OA) and CPPD [1]. Recurrent acute CPP arthritis and chronic recurrent CPP arthropathy can lead to severe joint destruction, sometimes very rapidly.

Currently, no specific treatment has demonstrated efficacy to prevent or slow CPP deposition [2]. Acute inflammation induced by CPPD crystals can be nonspecifically treated by nonsteroidal anti-inflammatory agents (NSAIDs), glucocorticoids or colchicine, but these symptomatic treatments are very often ineffective, particularly in recurrent or chronic cases [2]. Furthermore, these agents are frequently not tolerated or contraindicated in older patients, the population most frequently affected by chondrocalcinosis. More importantly, none of the available treatments proved able to stop gradual joint damage due to chondrocalcinosis. More recently, promising results have been reported with biologic anti-interleukin 1 (IL-1) agents [3,4], but these agents are extremely expensive and not reimbursed by most health systems or insurances for this indication.

At low doses, methotrexate (MTX) is a potent anti-inflammatory agent, considered the gold standard therapy for many inflammatory arthritides. MTX could conceptually be effective in CPP arthropathies since it interferes with glutathione metabolism, decreasing cell recruitment to the inflamed joint and promoting the release of the endogenous anti-inflammatory mediator adenosine and IL-1Ra [5]. Given its good safety profile and low cost, MTX could represent an interesting therapeutic option, particularly for patients resistant to classic anti-inflammatory treatments. Two case series suggested that MTX is a safe and effective therapeutic alternative for CPPD arthropathy refractory to standard therapy [6,7], but were contradicted later by a small French case series [8]. To date, no controlled study has examined the impact of MTX in refractory CPP arthropathy. We therefore conducted a randomized controlled trial to test the efficacy of MTX on symptoms and signs of chronic or recurrent CPP arthropathy.

## Methods

### Study design

**Figure 1:**
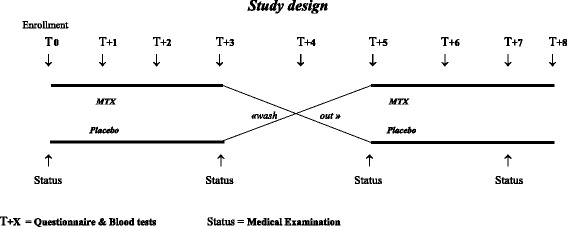
**Study design.** This was a double-blind, crossover randomized controlled trial. Patients were randomized to receive either methotrexate (MTX) or placebo (PBO) during an initial treatment period of three months, followed by a ‘wash-out’ phase of two months and a crossover second treatment period of three months. The T + X (↓) represent monthly assessments since randomization; the status (↑) represent medical visits with a clinical examination.

This was a double-blind, crossover randomized controlled trial. Patients were randomized to receive either MTX or placebo (PBO) by subcutaneous (sc) injection during an initial treatment period of three months, followed by a ‘wash-out’ phase of two months and a crossover second treatment period of three months (Figure 1). Period durations of three months’ treatment were selected to capture the relatively slow-acting anti-inflammatory effects of MTX and usual natural fluctuations of the disease. Once monthly, patients were examined, asked to fill out questionnaires and had routine blood tests.

### Study population and recruitment

We intended to study the subset of CPPD arthropathy patients presenting with recurrent clinical manifestations of acute mono- or oligoarthritis (‘pseudogout’), or persistent polyarthritis. We enrolled all consecutive patients from the Rheumatology Departments of several university hospitals (Geneva, Dublin, Zurich) and regional hospitals (La Chaux-de-Fonds and Yverdon) who met the inclusion criteria and accepted to enter the study. The inclusion criteria were:

- Definite CPPD disease using the McCarty diagnostic criteria [9].
- Recurrent mono- or oligoarthrits (‘pseudogout’) (at least three flares/six months) or persistent polyarthritis.
- Unsatisfactory response to at least one NSAID or low-dose glucocorticoids (defined by the patient), OR contraindication to NSAIDs and glucocorticoids (defined by the physician).

The exclusion criteria were:

- A contraindication to MTX treatment: hepatic failure, important alcohol consumption, severe renal failure, hematological disease, acute infection.
- A diagnosis of an alternative rheumatic condition: rheumatoid arthritis, connective tissue disease, psoriatic arthritis, gout or any other chronic or recurrent disease associated with oligo- or polyarthritis.
- Inability to fill out a questionnaire in the local language.

The study was approved by the local ethics committees (Geneva, Lausanne, Bern, Zurich, Switzerland and Mater Misericordiae University Hospital, Dublin, Ireland) and the Swiss federal health authorities (Swissmedic). Informed consent was obtained from all participants. The trial was registered (EudraCT N°: 2007-003479-37, ISRCTN79343755) [10].

### Randomization/blinding

Patients were randomized to either MTX or PBO in balanced blocks of six by means of a random number generator. The medication codes were kept in sealed envelopes until the end of the study. Both patients and the investigators were blinded to the treatment group assignment.

### Intervention

Patients were randomized to receive either MTX or PBO during an initial treatment period of three months, followed by a ‘wash-out’ period of two months, and a subsequent treatment period of three months with the alternative regimen. Patients were started on MTX therapy with 7.5 mg the first week, which was increased to 15 mg/week if tolerated. All patients also simultaneously received folic acid supplementation (5 to 10 mg/week) to reduce side effects, as recommended. Patients received their first sc MTX injection by a nurse and were taught how to perform sc injections themselves. Subsequent MTX injections were self-administered with commercially available pre-filled syringes (Metoject™, Gebro Pharma AG, Liestal, Switzerland) [11]. Identical pre-filled syringes containing NaCl 0.9% were produced and packed by the hospital’s pharmacy. New medications were provided at each visit and exchanged for unused medications, to assess compliance by syringe count.

Patients were allowed to use concomitant medications as judged best by their physicians, without modifications during the study (from three days before T0, to T +8). In addition, treatment of CPPD arthropathy flares with a three-day course of NSAIDs, a three-day course of oral glucocorticoids (≤30 mg/d of equivalent prednisolone) or a three-day course or increase of colchicine was allowed. Intra-articular injections of glucocorticoids or high doses of oral glucocorticoids (>30 mg/day) were not allowed.

### Outcome measures

The primary outcome was disease activity score based on 44 joints (DAS44). The DAS44 is a validated assessment tool of disease activity in rheumatoid arthritis (RA) that has been used in many other chronic arthritides. The DAS44 is a composite outcome measure including the number of swollen joints, the number of tender joints and the erythrocyte sedimentation rate (ESR).

Computation of the DAS44 [12]:

$$ \mathrm{D}\mathrm{A}\mathrm{S}44=0.54\surd \left(\mathrm{Tender}\ \mathrm{count}\right)+0.065\surd \left(\mathrm{Swollen}\ \mathrm{count}\right)+0.33\; \ln \left(\mathrm{E}\mathrm{S}\mathrm{R}\right)+0.007\left(\mathrm{patient}\ \mathrm{assessment}\right) $$

The number of tender and swollen joints was assessed by the physicians at the beginning and at the end of each treatment period during the medical examination. ESR was measured at regular intervals on each blood test. General health was evaluated using a Lickert scale ranging from 0 to 10 by the physician.

Secondary outcomes included the number of acute arthritis flares for the acute recurrent forms, and disease and pain levels (visual analog scale (VAS)) for the chronic forms, which have been shown to be sensitive to change in other arthritides

- ***Acute arthritis flares:*** Acute arthritis flares (‘pseudogout flares’) are an important outcome as these are one of the most easily recognized concerns of patients. However, flares have not yet been well defined in the arthritis literature [13]; we therefore only recorded the number of subjective arthritis flares and let patients judge what is or is not a ‘flare’ or an ‘attack’. We considered transient increases in concomitant glucocorticoids as a flare equivalent.
- ***Pain:*** Pain was measured using a VAS of the target joints.
- ***Safety:*** We monitored MTX side effects according to published guidelines [14]. All patients were required to have a recent blood test prior to enrollment to check liver function tests, hepatitis serology (<1 year), blood cells and renal function. A baseline chest X-ray was also performed. All patients had their liver function tests, blood cell count and creatinine tested at monthly intervals. Other potential side effects were monitored at monthly intervals. Among others, patients were specifically asked about nausea, vomiting, abdominal pain, diarrhea, cutaneous affections, mucous ulcerations, cough and dyspnea.
- ***Other secondary outcomes:*** Included patient’s global assessment, function of the target joints, ESR and serologic markers of inflammation (blood tests), duration of morning stiffness, number of tender and swollen joints, number of analgesic pills, cumulative dose of glucocorticoids, NSAIDs or colchicine and safety [13]. Patient’s global assessment of their general health was evaluated using a Lickert scale ranging from 0 to 10. Functional impairment were determined by asking the patient to assess function in the involved joints (3 = total disability, 2 = movement possible, 1 = weight bearing possible, 0 = painless full function [13]). We also recorded the length of morning stiffness in minutes, and number of analgesic, and anti-inflammatory pills taken during the previous week. The type of clinical presentation of CPPD arthropathy was based on the number of synovitic joints on the physical examination during the three-month treatment period: one swollen joint was classified as a monoarticular presentation, between two and four swollen joints as an oligoarticular presentation, and more than four swollen joints as a polyarticular presentation. The persistence of the clinical presentation was based on the assessment of the enrolling physician (presence of pseudogout flares) and the duration of patient’s physical impairment by his arthritides (>50% of days impaired classified as chronic arthropathy)**.**

### Analysis

We calculated the sample size based on published data on DAS44 changes in trials with MTX in RA, with the goal of being able to detect a difference at least as large as the effect size of a moderate clinical response (0.6 points, EULAR response criteria) [15]. Assuming a matched analysis (crossover design) and a type one error of 5%, 28 pairs would be needed to reject the null hypothesis with a statistical power of 80%.

Efficacy analyses were performed on the intent-to-treat basis. Patients lost to follow-up after enrollment were assumed not to have benefitted from the intervention and a last observation carried over procedure was used to impute the missing follow-up assessments. Individual questionnaire scores, measured at monthly intervals, were correlated and thus an analysis for repeated measure analysis was performed. It is not known precisely when the therapeutic efficacy of MTX emerges in CPPD arthropathy. Based on our pilot study [6], we estimated that the therapeutic efficacy would appear within the three first months after treatment initiation. In order to analyze the DAS44 responses over time for both groups (PBO and MTX), we use a marginal longitudinal model, which consists of modeling parametrically the marginal mean of the responses as well as the correlations between individuals. Since both groups are similar at baseline by design, the marginal expectation of the response depends only on a group effect (MTX vs. PBO), a quadratic time trend specific to each period (in order to capture the natural evolution over time), and the DAS44 at baselines (T0 and T +5, see Figure 1), and an interaction period - treatment group [16].

We tested the possibility of a carry-over effect for patients who received MTX as their first treatment by adding the treatment sequence into the model. We also analyzed the possibility of effect modification by type of CPPD arthropathy (monoarticular vs. polyarticular presentation). All analyses were conducted with the R statistical software (R version 2.15.2) [17].

## Results

**Figure 2:**
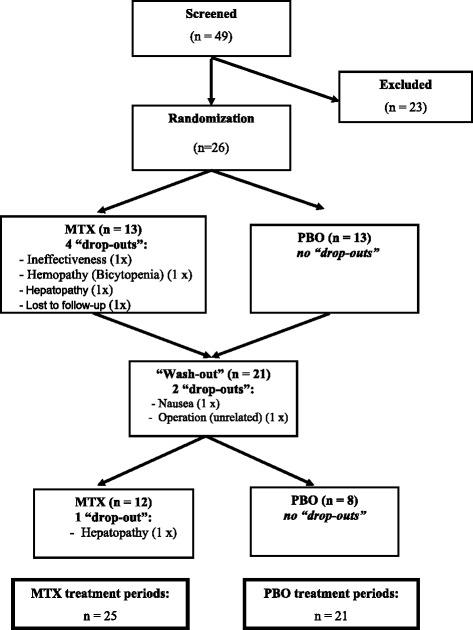
**Flow diagram of the study.** Displays the study flow: 49 patients were screened, 26 corresponded to the inclusion and exclusion criteria and signed the study informed consent form, 21 (81%) finished the first treatment period and 20 (95%) the second treatment period. Overall, 25 patients completed the methotrexate (MTX) treatment period and 21 the placebo (PBO) treatment period.

Forty-nine patients were screened for participation in the trial, and after a medical assessment, 26 patients were randomized (Figure 2). Seven patients did not complete the whole trial; the most common reason for discontinuation was a transaminase elevation above three times the normal values. The baseline characteristics at the initiation of the trial treatment were balanced (Table 1). Subjects ranged from 46 to 84 years (median = 71, IQR (54.5 to 76.0), mean = 66.4 ± 12.8) of age and were all Caucasian.

**Table 1:** **Baseline characteristics at the initiation of the respective treatment periods**

**Disease characteristics** ^**(1),(2)**^	**Treatment period MTX (N = 25)**	**Treatment period PBO (N = 21)**	***P***
Age [years], mean ± sd	62.8 ± 13.7	69.3 ± 11.9	0.374
Male sex [%]	44.4	36.4	0.754
Number of tender joints^(3)^, median	4.5 [2.75 – 12.0]	5.5 [2.0 – 9.25]	0.465
Number of swollen joints^(4)^ , median	2.5 [1.0 – 4.75]	2.0 [1.0 – 9.5]	0.782
CRP^(5)^ [mg/l], median	3.0 [2.0 – 7.0]	3.15 [2.0 – 6.93]	0.632
DAS44^(6)^, median	2.5 [1.7 – 3.0]	2.1 [1.7 – 3.1]	0.957
Pain - VAS^(7)^, median	6 [4.75 – 8.25]	6 [5 – 7]	0.270
Physician global assessment^(8)^, median	4.5 [3 – 6.25]	5 [4 – 6]	0.311
Number of analgesics/week^(9)^, median	13.5 [2.0 – 28.0]	10.0 [2.5 – 26.0]	0.684
Number of self-reported flares^(10)^, median	1 [0 – 2]	1 [0 – 1]	0.763
Clinical presentation^(11)^ [N (%)]			
Monoarticular	3 (12%)	2 (10%)	0.957
Oligoarticular	10 (40%)	9 (43%)	
Polyarticular	12 (48%)	10 (48%)	
Persistence of symptoms^(12)^ [N (%)]			
Recurrent arthritides (‘pseudogout’)	4 (22%)	7 (39%)	0.471
Chronic arthritides	14 (78%)	11 (61%)	

^(1)^Since this is a crossover trial, patients can contribute both to the MTX and the PBO arm. Patient and disease characteristics are compared at the initiation of the respective treatment periods, which could either be at T0 or T +5 on Figure 1. ^(2)^Values are given in mean (standard deviations (sd)), in medians (interquartile ranges), or in absolute numbers (proportions), as indicated. ^(3)^The number of tender joints could range from 0 to 44; ^(4)^the number of swollen joints could range from 0 to 44; ^(5)^C-reactive protein ranged from 0 to 28, with the normal range being <10; ^(6)^the disease activity score based on 44 joints rages from 0 to 10; ^(7)^the visual analog scale for pain ranges from 0 to 10; ^(8)^the physician global assessment scale ranges from 0 to 10; ^(9)^patients reported the mean number of analgesic pills taken during the previous week, ranged from 0 to 50; ^(10)^number of self-reported arthritic flares during the previous month; ^(11)^the clinical presentation was based on the maximum number of swollen joints during the three- month treatment period; ^(12)^the persistence of symptoms was based on the assessment of the enrolling physician. This information was not always recorded (missing in eight patients). MTX, methotrexate; PBO, placebo; CRP, C-reactive protein; DAS44, disease activity score based on 44 joints; VAS, visual analog scale.

**Figure 3:**
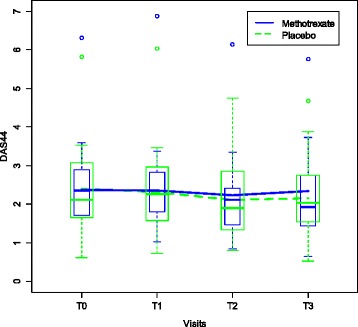
**Evolution in disease activity.** Displays the evolution of the disease activity as measured by the disease activity score based on 23 joints (DAS28) while receiving either methotrexate (MTX) (N = 25) or placebo (PBO) (N = 21). This figure aggregates both treatment periods, before and after the crossover.

Disease activity, as measured by the DAS44 improved over time (*P* = 0.005) but was not significantly improved by treatment with MTX. As for the secondary outcomes, none of the other secondary outcomes improved significantly by treatment with MTX (Table 2, Figure 3).

**Table 2:** **Change in outcomes between start of treatment and three months**

**Outcomes**	**Treatment course: MTX**	**Treatment course: PBO**	***P*** **value**
	**(N = 25)**	**(N = 21)**	
**DAS44**	−0.08 [−0.6, 0.1]	−0.13 [−0.6, 0.1]	0.44
**Number of tender joints [0 – 44]**	0 [−1.0, 1.3]	−1 [−3.5, 0.5]	0.17
**Number of swollen joints [0 – 44]**	−1 [−3.5, 0]	0 [−1.5, 2.0]	0.56
**CRP level**	0.2 [−0.8, 1.9]	−0.3 [−1.6, 1.6]	0.33
**Number of analgesic pills/week, median**	0 [−7, 4]	0 [−2.0, 1.5]	0.36
**Number of flares/3 months**	0 [−1, 0]	0 [−1, 0]	0.10
**VAS Pain**	−1 [−2.8, 0]	0 [−2, 1]	0.43

All results are displayed in medians [interquartile ranges]. MTX, methotrexate; PBO, placebo; DAS44, disease activity score based on 44 joints; CRP, C-reactive protein; VAS Pain, visual analog scale for pain.

We also examined the possibility of an effect modification by the type of CPPD arthropathy (monoarticular presentations vs. polyarticular presentations) and found no evidence that MTX was more effective in patients with polyarticular presentation (test for effect modification, *P* = 0.27).

During the study follow-up, 35 adverse events (AEs) were reported, 16 on MTX and 18 on PBO (*P* = 0.81) and 1 during the wash-out period. However, more patients dropped out on MTX than on PBO (5 vs. 0), mostly for common AE related to MTX. One serious AE was observed on a patient on methotrexate: an acute bicytopenia, which imposed hospitalization, fortunately without permanent damage.

## Discussion

This randomized controlled, crossover trial was conducted to assess the efficacy of MTX in chronic CPPD arthropathy. We found that after three months of treatment, 15 mg of sc MTX, did not significantly improve the symptoms of CPPD disease, while MTX was associated with increased AEs typically related to this therapy. To our knowledge, this is the first PBO-controlled trial to investigate the efficacy of MTX, an inexpensive and well-tolerated anti-rheumatic drug, in patients with chronic CPPD arthropathy.

This controlled study therefore contradicts our preliminary observational study as well as another Spanish report [6,7]. Several hypotheses can explain the contradiction between favorable observational findings and a negative trial. First, this trial was possibly underpowered, leading to the possibility of a false negative result. Indeed, it proved extremely difficult to set up a multicenter, multinational, investigator-based study allowing the recruitment of enough patients. However, because of the crossover design, the trial still had a predicted power of 74% to detect a moderate change in DAS44, the study’s primary outcome. While the possibility of a false negative result exists, the likelihood that MTX has a large beneficial effect on the whole spectrum of chronic CPPD arthropathies is low. Furthermore, our outcome measures might not have been adequate to capture a potential beneficial effect of MTX; however, currently no validated outcome measures are available for the different clinical presentations of chronic CPPD arthropathy. The fact that MTX did not demonstrate a significant effect on the primary outcome, nor any of the five secondary outcomes, strongly suggests that this is not a false negative result. Another possible explanation for this finding is that we included patients not necessarily during a flare, with baseline disease activity too low (DAS44 between 2 and 3, median CRP around 3) to demonstrate a large effect size. Furthermore, as the natural course of the disease is one of flares and spontaneous improvements, a phenomenon of regression to the mean, makes the demonstration of a beneficial treatment effect even more difficult. This phenomenon could be amplified by the fact that flares frequently occur at intervals longer than three months, especially in patients with early disease.

In addition, CPPD disease is notoriously heterogeneous [1] and differential responses to a given medication are likely. This study has included patients with a variety of CPPD arthropathy clinical presentations (chronic or recurrent arthropathies, mono-, oligo- or poly-articular), which may have further diluted the study’s ability to demonstrate an effect of MTX. Another major difficulty is the fact that this aged population is frequently affected by multiple coexisting pathologies, including osteoarthritis and other seronegative arthritides, which can potentially mask a beneficial therapeutic effect. In addition, induction of transient arthralgias in some patients is a well-recognized adverse effect of MTX [18], which could have further obscured a potential beneficial effect. Finally, our study design allowed patients to take auto-medication of analgesics, NSAIDs and low-dose glucocorticoids, which may further have obliterated our capacity to demonstrate a potential treatment effect of MTX.

The crossover design assumes that the effect of the medication is transitory and removed quickly after treatment discontinuation during the ‘wash-out period’, with no carry-over effect thereafter. The pharmacological half-life of MTX is typically around four to six weeks in healthy individuals, thus a two-month wash-out period appears adequate. We examined a possible carry-over effect in the analysis, but did not find any statistically significant one. We cannot exclude the possibility that a potential beneficial effect of MTX might have appeared after three months. It is possible also that a dose of 15 mg of sc MTX was insufficient for some of the patients; it was selected because this dose of MTX has proved to be effective in older RA patients [11], and in our preliminary study, but possibly a higher dosage could have been more effective. The compliance of the study medication was good, as the sc injections were either performed by a nurse, at least in the beginning, and when not, all syringes were verified after use to control proper emptying. No specific outcome measures have been validated for CPPD arthropathy, which forced us to use instruments developed and validated for other rheumatic conditions. However, the clinical presentation of complicated CPPD is often similar to other common rheumatic conditions, such as gout or seronegative inflammatory arthritis, which should make the use of outcome measures for these diseases good instruments for chondrocalcinosis as well.

Famous precedents actually exist on the sometimes wide discrepancies between observational and controlled studies, which can be explained by designs of the later ones that afterward appeared unsuitable to demonstrate an effect [19]. We therefore strongly believe that further studies involving more centers and patients, a better selection of the cases, if possible with less comorbidities, longer treatment periods and a more strictly defined intake of other medications is warranted.

## Conclusions

Chondrocalcinosis is indeed one of the most common joint disorders, affecting up to 5% of the human population, with the prevalence rising to 15% over 60 years of age [20]; its incidence will continue to rise because of the increasing age of the population. CPPD disease is certainly under-recognized, often misdiagnosed and frequently only treated symptomatically, which can lead to accelerated joint destruction. Existing treatments are often unsatisfactory and currently no anti-rheumatic therapy has been formally tested in CPPD arthropathy. The biologic anti-IL-1 agents are promising for some patients, but certainly do not represent an option for a vast majority of patients throughout the world at least for a while, in particular because of their price. Effective, safe and cheap therapeutic options are therefore clearly needed to improve the quality of life of patients not responding to simple NSAID therapy. At this time, in view of this study and other reports [8], MTX is clearly not a treatment solution for all patients with chronic CPPD arthropathy. However, some patients continue to take MTX because of strong perceived benefits, after being informed of their treatment arm. In addition, MTX may have the potential to reduce joint damage in chronic CPPD arthropathy, given its pharmacological properties and established protective effects in RA [5], and this might be considered as an outcome in future studies. We therefore believe that MTX, despite this negative study, is still an option for selected patients with inadequate response or intolerance to the other available medications, and that further studies are warranted.

## Abbreviations

AE: adverse event
CPPD: calcium pyrophosphate deposition
CRP: C-reactive protein
DAS44: disease activity score based on 44 joints
ESR: erythrocyte sedimentation rate
IL-1: interleukin 1
MTX: methotrexate
NSAIDs: nonsteroidal anti-inflammatory drugs
OA: osteoarthritis
PBO: placebo
RA: rheumatoid arthritis
sc: subcutaneous
VAS: visual analog scale
